# On the Use of Creasote in Diarrhea

**Published:** 1852-04

**Authors:** T. M. Woodson

**Affiliations:** Sumner county, Tenn.


Art. II.—*On the Use of Creasote in Diarrhea.* By T. M. Woodson, M. D., of Sumner county, Tenn.

In the "American Journal of the Medical Sciences" for July, 1851, is a notice of the use of *creasote* in diarrhea, by Mr. Kestevan, whose testimony in its favor is very strong. He states, in fact, that in his hands the remedy has never failed, and that generally he found a single dose sufficient. His dose was from one to five minims. Reading the statement of Mr. Kestevan at a season when diarrhea was rife, I determined to give the remedy a trial. The first case in which I administered it occurred about the first of August. The subject was a negro woman aged thirty-five years, who had been affected for two weeks before medical advice was solicited. I at first prescribed opium and tannin, but as they afforded only temporary relief, I was requested to visit her on the 4th of

August. I found her in the following condition: Pulse not unnatural, and skin rather cool and moist; tongue coated with a white fur; patient complains of severe pain in the abdomen, which is increased by pressure; has from twelve to twenty serous evacuations daily and is much prostrated. I prescribed creasote, one drop every four hours, in combination with camphorated tincture of opium and aromatic spirits of ammonia.

Aug. 5th. Patient much improved this morning; bowels were moved but twice after the first dose of the medicine —has taken two doses. She continued to improve, and soon recovered.

*Case* 2.—A female child, aged fourteen months, was taken with diarrhea on the 11th of August. Vomiting also attended. I saw the case the following morning, and found the child laboring under some fever, with great derangement of the stomach and bowels. Prescribed calomel and Dover's powder, which were to be repeated every two hours. I was called at midnight again, and found the symptoms rather aggravated than improved. I at once administered creasote, half a drop, with tinct. opi. camph. and spirits ammon. aromat., and repeated every three hours. The patient was relieved of its distressing nausea and diarrhea before morning, and had a prompt recovery.

*Case* 3.—A little boy, aged five years, was seized on the 11th of August, with symptoms similar to those presented by the last case. Without any preliminary measures, I ordered in this case the creasote combined as before, beginning with half a drop and increasing it to a drop at a dose. In ten hours the disorder was checked, and the patient was soon as well as ever.

*Case* 4.—J. D. C., aged fifteen years, had labored under diarrhea for three weeks when he applied for medical advice. He was at that time much debilitated, and his complaint appeared to be progressive. I prescribed the creasote mixture, every four hours, directing the creasote to be increased after a few doses to two drops. Soon after taking the first dose he had two evacuations in quick succession, but the diarrhea then ceased, and in a few hours he was comfortable.

*Case* 5.—L. H., aged fifty years, has been for many years subject to diarrhea every summer. He has from eight to ten evacuations daily, and has become much debilitated. He was promptly relieved by the creasote mixture, and always found relief from it when the symptoms returned during the hot season.

*Case* 6.—Miss C., aged nineteen years, has been subject to diarrhea just before her catamenial periods. It is profuse and exhausting, continuing generally for about ten days. The most prompt relief was derived from the creasote.

I have used this article in more than twenty cases of diarrhea with similar results, but it would be tedious to recite others. I have found it much more prompt and invariable in its action than any other remedy to which I have ever resorted in this complaint, and, although it is described by writers on materia medica as an irritant and narcotic, I do not hesitate to administer it where fever, and dysenteric symptoms are present. It is superior to opiates, in that it does not leave the bowels constipated. It excites no nausea, nor any other unpleasant symptom. Its action seems to be stimulant and antispasmodic, allaying pain very promptly, and imparting to the patient an agreeable sense of warmth in the stomach and bowels. Whether it acts directly as an astringent, or, as suggested by Mr. Kestevan, by coagulating the albuminous fluids of the alimentary canal, I do not pretend to decide. But of its efficiency in bowel complaints, I think there can be no question, and in such cases it is confidently recommended.

Feb. 25th, 1852.

